# The economic impact of mental healthcare consumption before and after stroke in a cohort of stroke patients in the Netherlands: a record linkage study

**DOI:** 10.1186/s12913-016-1915-3

**Published:** 2016-12-13

**Authors:** M. van Eeden, G. A. P. G. van Mastrigt, S. M. A. A. Evers, E. P. M. van Raak, G. A. M. Driessen, C. M. van Heugten

**Affiliations:** 1 Department of Health Services Research, CAPHRI, Research School for Public Health and Primary Care, Faculty of Health, Medicine and Life Sciences, Maastricht University, P.O. Box 616, 6200 MD Maastricht, The Netherlands; 2 MHeNS, School for Mental Health & Neuroscience, Department of Psychiatry & Psychology, Faculty of Health Medicine and Life Sciences, Maastricht University, Maastricht, The Netherlands; 3 Department of Neurology, Maastricht University Medical Centre, Maastricht, The Netherlands; 4 Department of Neuropsychology & Psychopharmacology, Faculty of Psychology & Neuroscience, Maastricht University, Maastricht, The Netherlands

**Keywords:** Stroke, Healthcare consumption, Economic impact of stroke, Record linkage study

## Abstract

**Background:**

Post-stroke healthcare consumption is strongly associated with a mental health diagnosis. This study aimed to identify stroke patients who utilised mental healthcare facilities, explored their mental healthcare consumption pre-stroke and post-stroke, and examined possible predictors of costs incurred by mental healthcare consumption post-stroke.

**Methods:**

Three databases were integrated, namely the Maastricht University Medical Centre (MUMC) Medical Administration, the Stroke Registry from the Department of Neurology at MUMC, and the Psychiatric Case Registry South-Limburg. Patients from the MUMC who suffered their first-ever stroke between January 1 2000 and December 31 2004 were included and their records were analysed for mental healthcare consumption from 5 years preceding to 5 years following their stroke (1995–2009). Regression analysis was conducted to identify possible predictors of mental healthcare consumption costs.

**Results:**

A total of 1385 patients were included and 357 (25.8%) received services from a mental healthcare facility during the 10-year reference period around their stroke. The costs of mental healthcare usage increased over time and peaked 1 year post-stroke (€7057; 22% of total mental healthcare costs). The number of hospitalisation days and mental healthcare consumption pre-stroke were significant predictors of mental healthcare costs. Explained variances of these models (costs during the 5 years post-stroke: *R* ^2^ = 15.5%, costs across a 10 year reference period: *R* ^2^ = 4.6%,) were low.

**Conclusion:**

Stroke patients have a significant level of mental healthcare comorbidity leading to relatively high mental healthcare costs. There is a relationship between stroke and mental healthcare consumption costs, but results concerning the underlying factors responsible for these costs are inconclusive.

## Background

Chronic diseases, such as stroke, are by far the leading cause of mortality and permanent disability worldwide. Recent data from the World Health Organization (WHO) indicate that worldwide 1 out of every 466 people suffer a stroke each year (15 million in total) of which one third dies and one-third is permanently disabled [1]. In 2012 in the Netherlands, 1% of the population suffered from a stroke and almost 7% of this group died due to stroke complications [2]. As treatments for acute stroke advance, the number of stroke survivors, and hence people living with a permanent disability, is likely to increase in the future.

Post-stroke healthcare consumption is strongly associated with a mental health diagnosis [3]. A prospective study on the association between psychiatric and cognitive symptoms post-stroke showed that patients with psychiatric symptoms after a stroke are at an increased risk of developing cognitive deficits and a decline in cognitive function, and are therefore more likely to use mental healthcare facilities [4]. In addition, various studies have found a high incidence of post-stroke depression leading to increased mental healthcare consumption [5–8].

Not only is the impact of stroke on mental health significant, the economic impact of stroke is also considerable. Currently, approximately 3–4% of the total healthcare budget in Western countries is spent on stroke patients [9]. In the Netherlands, costs of general mental healthcare increased from 5.9% (€2.6 billion) of the total healthcare expenditures to 7.3% (€5.4 billion) [10]. In 2001, Evers et al. explored the post-stroke use of mental healthcare facilities in the Netherlands by conducting a study in which two healthcare databases were linked for the period from 1987 to 1995 [11]. They showed that stroke and mental healthcare costs are significantly related (1.3% of total mental healthcare costs were induced by stroke patients), but that further research into the predictors of stroke-related mental healthcare costs is necessary.

Although recent evidence on stroke and mental healthcare costs is limited, there is a wide variety of studies on stroke-related costs in terms of population, methods, disease severity, cost categories and perspective [12]. Demographic, patient and stroke-specific characteristics, such as age [13] sex [14], length of stay [14, 15], country [16], Modified Ranking Scale (MRS) scores [17], stroke type [16–18] and stroke severity [14] have all proven to be significant predictors of stroke related costs. In addition, potential risk factors for stroke such as hypertension [19], diabetes [15, 19], and smoking [19] were found to be significant predictors of stroke-related costs. Biological characteristics such as heart failure [14], hyponatraemia [15], and atrial fibrillation [14], also appeared to be significant predictors of costs.

Due to the aging of the population and a growing demand for stroke care, stroke-related healthcare costs are likely to increase in the future. For governments, this causes discrepancies between cutting stroke-related healthcare costs on the one hand and dealing with the increasing demand for stroke-related healthcare interventions on the other hand. Therefore, it is imperative to gain more insights into the costs associated with stroke, which can be used to make valid recommendations and suggestions for healthcare policy makers and support them in their decisions to adopt or reject new healthcare technologies. Over the last decade, evidence of the costs incurred by mental healthcare consumption related to stroke in the Dutch context has been limited. And previous research concerning the Dutch context is out-dated [9, 11], creating a strong need for up-to-date evidence. In gathering this evidence, it would be helpful to identify possible predictors for mental healthcare consumption. The aim of this study was to identify stroke patients who use mental healthcare facilities, to explore this consumption 5 years pre-stroke and 5 years post-stroke, and to examine possible predictors of costs incurred by mental healthcare consumption after a stroke.

## Methods

### Design

The current study is a record linkage study in which three separate databases were integrated. The Medical Ethics Committee of Maastricht University (METC AZM/UM) approved the current study. Since this study used data from registers and databases and did not involve interaction with patients, the METC AZM/UM confirmed that no informed or written consent was necessary for this study.

### Setting

The current study took place in the city of Maastricht (120,000 inhabitants), a relatively small city in the south of the Netherlands (Limburg province), and its surrounding area (80,000 inhabitants). The Maastricht University Medical Centre (MUMC) is the only hospital in this catchment area of 200,000 inhabitants with both a regional and a top-referral care function. In the MUMC, all patients are registered with an ICD-9 or an ICD-10 classification (ICD: International Statistical Classification of Diseases and Related Health Problems), depending on the year of admission.

### Databases

Three databases were integrated in this study: the Maastricht University Medical Centre Medical Administration (MA), the Maastricht Stroke Registry (MSR) and the Psychiatric Case Registry South-Limburg (PCR). The MA is the primary registry of the MUMC, and all patients admitted to the hospital are registered with an ICD-9 or an ICD-10 classification. The MSR is the prospective database from the Department of Neurology at the MUMC, which contains the clinical, functional and outcome data from all adult stroke patients (over 18 years of age) who have been treated by the department since July 1 1987. The PCR has been used since 1981 as a prospective registry for collecting data from all patients who use mental healthcare facilities in the Maastricht region.

Demographic and patient characteristics such as age, gender, living with a partner, and discharge location were collected from the MA. The MSR and clinical records were used to collect possible predictors for stroke-related mental healthcare costs, such as type of stroke (ischaemic or haemorrhagic; cortical or lacunar), Modified Ranking Scale (MRS) score, history of Transient Ischaemic Attack (TIA), hypertension, smoking, alcohol use and diabetes. In addition, information on patient contact with mental healthcare facilities in terms of day-treatment, clinical and outpatient care, and assisted living, was derived from the PCR.

### Timeline and inclusion criteria

The year 2009 was the last year in which complete data were available in the PCR database. Therefore, it was decided to include adult patients with ICD-9 code 430-438 or ICD-10 code 160-169 who suffered a stroke between January 1 2000 and December 31 2004, which allowed us to analyse patient data from 1995 until 2009 (analyses from 5 years before the first stroke case until 5 years after the last stroke case). Patients were included if they suffered their first ever stroke in the period between 2000 and 2004. Patients were excluded if they suffered from recurrent stroke, a subarachnoid haemorrhage, a TIA or another vascular non-stroke event such as an asymptomatic carotid stenosis, if they appeared to live outside the catchment area (based on area codes); and if death occurred during hospital admission for the first-ever stroke.

### Linkage

The MA and MSR were linked which resulted in a complete database of stroke patients who suffered their first-ever stroke between January 1 2000 and December 31 2004. Based on patient characteristics, a unique anonymous code was constructed for each individual patient. Through these codes, patients from the stroke databases were identified in and linked to the PCR database. The linkage was based upon the first letter of the family (maiden) name, gender and date of birth. Experience from previous research with the PCR showed that these identifiers gave the best linkage, by minimising the chance of mistaken identity [20].

### Cost analysis and valuation

Costs were calculated based on quantities derived from the PCR. Four mental healthcare costs categories were included: costs of day-treatment, costs within the clinical setting, costs incurred in the outpatient clinic and costs of assisted living. Costs of day-treatment are based on the costs of a psychiatric treatment day in a university hospital. Clinical setting costs consisted of the costs of visits to a general psychiatric hospital, the psychiatric department of a university hospital and the psychiatric department of a nursing home. Outpatient clinic costs were based on visits to the outpatient department of a psychiatric or university hospital and contacts with a psychotherapist. Costs of assisted living were valued as the costs of assisted living arrangements, such as housekeeping, meals, and other assistance as needed. The updated Dutch manual for cost analysis in healthcare research was used for the valuation of these healthcare costs [21].

### Statistical analysis

The statistical package SPSS version 20 was used to merge all databases and to conduct all analyses. Missing data regarding resource use were handled through multiple imputations, a preferred technique for analysing data sets with missing values. It involves replacing each missing data point with a set of plausible values to generate complete data sets [22]. Independent samples *T*-test and Chi Square Tests were used to explore differences between the users of mental healthcare (PCR+) and nonusers of mental healthcare (PCR-). Significance was determined at the 0.05 level. Cost data were skewed, and therefore all cost values were logarithmically transformed to achieve a more normal distribution and permit the use of linear regression. A number of ‘0.00001’ was added to all cost values to avoid zero values for log transformation [23]. Two different linear regression models were constructed; one to estimate possible predictors of the mental healthcare costs post-stroke (Model 1) and a second model to identify possible predictors of the mental healthcare costs in the 5 years pre-stroke and the 5 years post-stroke (Model 2). To avoid over fitting of the model, one independent variable was selected as a predictor in the models for every ten patients who were included. Variables were entered into a linear regression analysis using backward stepwise selection, in which variables were removed from the model based on the probability of the likelihood statistics [23]. Selection of the prediction variables was based on the results from previous research, where predictors of high (mental healthcare) costs in a stroke population were identified [14–19]. Included variables were: sex, age, living with partner, number of hospitalization days, discharge location, stroke type (ischaemic or haemorrhagic, cortical or lacunar), Modified Ranking Scale (MRS) score, hypertension, diabetes, alive for at least 5 years post-stroke, and costs pre-stroke (last predictor was only used in regression model 1). Binary and continuous variables were included in the regression analysis, and dummies were created for the categorical variable discharge location (discharged to nursing home, discharged to home and discharged to rehabilitation centre). Due to the large amount of missing data, the variables TIA (yes/no), smoking (yes/no) and alcohol use (yes/no) were excluded from the regression models to avoid under fitting. These models were chosen, because they provide the most valuable information for answering the main research questions. Again, significance was determined at the 0.05 level.

## Results

### Subjects and selection

**Fig. 1:**
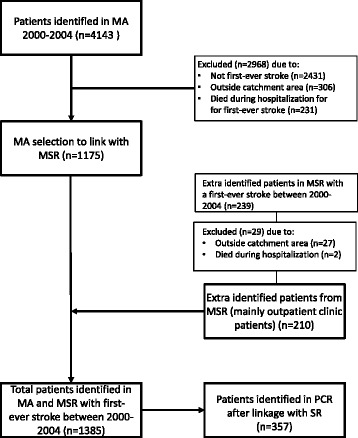
Identification of eligible patients with respect to the MUMC Medical Administration (MA), Maastricht Stroke Registry (MSR) and the Psychiatric Case Registry (PCR)

A total of 4143 patients who were given an ICD-9 or an ICD-10 stroke-related classification, between January 1 2000 and December 31 2004, were identified in the MA. Due to various reasons, 2968 patients were excluded, leaving 1175 patients for this study. An additional 210, mostly outpatient clinic patients were identified in the MSR resulting in a total of 1385 patients who suffered a first-ever stroke during the 2000–2004 period. Of this group of patients, 25.8% (*n* = 357) received services from a mental healthcare facility at any time within the 10-year reference period (5 years pre-stroke, and 5 years post-stroke). Further details are presented in Fig. 1.

### Psychiatric case registry (PCR)

To examine differences, stroke patients who were identified in the PCR (*n* = 357), further referred to as PCR+, were compared with patients who were not identified in the PCR (*n* = 1028), further referred to as PCR-. Significantly more patients were clinically admitted to the hospital in the PCR+ group, on average 6.5% more (*p* = 0.022). Also, patients in the PCR+ group spent on average four more days (*p* = 0.000) in the hospital post-stroke, which is significantly higher than those in the PCR- group. Further details on the differences in demographic and stroke characteristics between PCR+ and PCR- patients are presented in Table 1.

**Table 1:** Characteristics of patients identified in the Psychiatric Case Registry versus patients not identified in the Psychiatric Case Registry

		PCR+ (*n* = 357)		PCR- (*n* = 1028)	*p*-value^†^
Demographic characteristics	n.		n.		
Mean age in years at stroke onset (SD^*^)	357	69.0 (14.0)	1028	71.3 (12.1)	0.051
Gender (% male)	357	48.2	1028	49.1	0.762
Living with partner (%)	357	42.0	1028	42.4	0.903
Hospital admission (% clinical)	357	75.1	1028	68.6	0.022^‡^
Hospitalization days (SD)	320	19.2 (20.6)	856	15.2 (13.1)	0.000^‡^
* Discharge location*					
Nursing home (%)	357	35.9	1028	33.7	0.376
Home (+ rehabilitation) (%)	357	52.1	1028	54.0	0.631
Rehabilitation centre (%)	357	4.2	1028	5.4	0.392
Stroke characteristics					
Stroke type (Ischaemic,%)	357	82.1	1028	85.3	0.148
MRS^a^ score (SD)	331	3.3 (1.2)	938	3.2 (1.2)	0.103
History of TIA^b^ (% yes)	357	9.5	1028	11.2	0.298
Hypertension, history/now (% yes)	357	47.1	1028	51.3	0.143
Smoking, history/now (% yes)	357	34.4	1028	28.2	0.063
Alcohol use, history/now (% yes)	357	26.9	1028	21.4	0.072
Diabetes, history/now (% yes)	357	17.8	1028	17.1	0.568

^*^SD standard deviation

^a^ *MRS* Modified Ranking Scale

^b^ *TIA* Transient Ischemic Attack

^†^Non-parametric Mann-Whitney test or Chi-Square test used to test for significant differences between PCR+ and PCR-

^‡^Significant at the 0.05 level

### Mental healthcare consumption

**Fig. 2:**
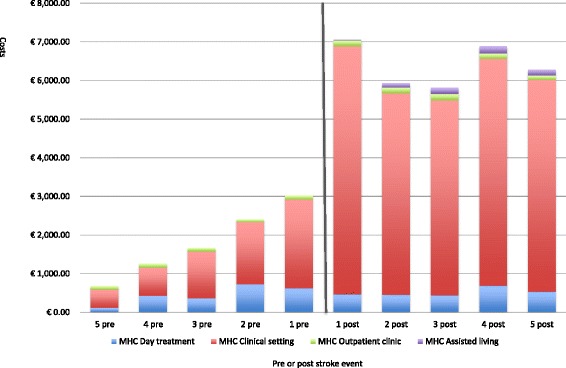
Total average mental health care consumption (MHC) 5 years before, and 5 years after stroke, split out in four cost categories (*n* = 357)

Figure 2 presents the average mental healthcare consumption 5 years pre-stroke and 5 years post-stroke. When comparing these periods, the costs of mental healthcare usage were higher post-stroke, with a peak in the first year post-stroke (€7057; 22% of total mental healthcare costs post-stroke). Total costs in 5 years pre-stroke were €9038 (with an average €1807 per patient per year) and total costs in 5 years post stroke were €31,966 (with an average €6393 per patient per year). In total, these costs increased by €22,928 per patient from the pre-stroke period to the post-stroke period. Day-treatment costs increase on average by €308; clinical setting costs increases on average by €21,734; outpatient clinic costs increased on average by €227; and assisted living costs increased on average by €660. Figure 2 also illustrates that there was an increase in mental healthcare costs over the 5 years pre-stroke. From the first year to the fifth year pre-stroke, the annual costs increased by €2344. When looking at the 5 years post-stroke, the annual costs decreased by €774, from €7057 in the first year to €6284 in the fifth year post-stroke.

Figure 2 also shows that costs within the clinical setting is by far the largest cost category, in both the 5 years pre-stroke (69.8%) and the 5 years post-stroke (87.7%). A total of 10,413 mental healthcare contacts took place during the 5 years pre-stroke, of which 5316 (51.1%) can be assigned to assisted living. In the 5 years post-stroke, a total of 39,958 mental healthcare contacts took place, of which 32,251 (80.7%) can be assigned to the assisted living. In addition, the top 25% of patients (*n* = 90) with the highest pre-stroke costs accounted for over 41% of the total post-stroke costs.

### Predictors of costs

Two regression models were built; one to identify possible predictors for total mental healthcare costs post-stroke (Table 2; model 1), and one for total costs pre- and post-stroke (Table 3; model 2). A total of 229 patients were included in both models. The R^2^ in model 1 was 0.155, meaning that 15.5% of the total costs post-stroke can be explained by the predictors included in the model. The results show that a higher age (*p* = 0.018, β =0.048) and more hospitalization days (*p* = 0.025, β = 0.026) led to significantly higher post-stroke costs. In addition, patients who used mental healthcare facilities preceding their stroke incurred significantly higher post-stroke costs (*p* = 0.000, β = 0.249) in comparison with patients without a history of mental healthcare consumption.

**Table 2:** Predictors of total post-stroke mental healthcare costs (Model 1)

Predictors^a^	Level	*β*	*p*	95% Confidence Interval
Living with partner	Yes/no	0.536	0.277	-0.433, 1.505
Age	Continuous	0.048	0.018^†^	0.008, 0.087
Number of hospitalization days	Continuous	0.026	0.025^†^	0.003, 0.049
Discharged to nursing home	Yes/no	0.364	0.751	-1.891, 2.619
Discharged to home	Yes/no	0.983	0.357	-1.196, 3.162
Stroke type	Ischemic/haemorrhagic	0.342	0.607	-0.968, 1.653
Stroke location	Left/right	0.506	0.267	-0.390, 1.403
MRS	Continuous	0.390	0.540	-0.861, 1.641
Hypertension	Yes/no	-0.277	0.545	-1.180, 0.625
Diabetes	Yes/no	-0.587	0.290	-1.679, 0.504
Alive within 10-year ref/period	Yes/no	0.956	0.060	-0.043, 1.954
Mental healthcare costs before stroke	Continuous	0.249	0.000^†^	0.118, 0.380
*Explained variance*	*0.155 (R* ^*2*^ *)*			

^†^Significant at the *p* = 0.05 level

^a^Predictors with only two levels or continuous predictors were included. Based on the likelihood method, the variable ‘sex’ was excluded

**Table 3:** Predictors of total pre-stroke and post-stroke mental healthcare costs (Model 2)

Predictors^a^	Level	*β*	*p*	95% Confidence Interval
Sex	Male/female	-0.505	0.135	-1.169, 0.158
Living with partner	Yes/no	0.756	0.039^†^	0.040, 1.472
Age	Continuous	0.016	0.263	-0.012, 0.043
Number of hospitalization days	Continuous	0.007	0.386	-0.009, 0.024
Discharged to nursing home	Yes/no	1.074	0.191	-0.539, 2.688
Discharged to home	Yes/no	1.087	0.169	-0.466, 2.640
Stroke type	Ischemic/haemorrhagic	0.210	0.658	-0.722, 1.142
Stroke location	Left/right	0.297	0.365	-0.348, 0.942
Diabetes	Yes/no	-0.214	0.589	-0.994, 0.566
Alive within 10-year ref/per	Yes/no	0.136	0.705	-0.569, 0.640
*Explained variance*	*0.047 (R* ^*2*^ *)*			

^†^Significant at the *p* = 0.05 level

^a^Predictors with only two levels were included. Dummy variables were created for predictors with more than two levels: Discharge location and MRS scores. Based on the likelihood method, excluded variables were: MRS, Hypertension

In model 2, the R^2^ is 0.047, indicating that only 4.7% of the variance of total pre- and post-stroke costs can be explained by the independent variables. However, one independent variable was significant at the *p* = 0.05 level. If patients were living with a partner they incurred significantly more costs (*p* = 0.039, β = 0.757) in comparison with patients who lived alone.

## Discussion

This study was conducted because of the need for new, up-to-date evidence regarding post-stroke mental healthcare consumption related costs in the Netherlands. The results of this study show that mental healthcare costs increase in the years preceding a first-ever stroke and that the majority of costs are incurred in the first year post-stroke. The results of factors contributing most to the high costs of stroke are inconclusive.

In this study, 25.8% (*n* = 357) of first-ever stroke survivors used mental healthcare facilities within the reference period of 10 years. On average, patients identified in the PCR (PCR+) spent 4 days more in the hospital than patients not identified in the PCR (PCR-). This might be explained by the fact that mental health comorbidity during a stroke-related hospital stay often leads to longer hospitalization. Mental healthcare consumption costs incurred within the clinical setting, outpatient costs, costs of day-treatment, and costs of assisted living increase significantly after a stroke. In general, costs increased from 5 years pre-stroke to 5 years post-stroke with €22.928 per patient, with an average total costs of €9038 in the 5 years pre-stroke and €31,966 in the 5 years post stroke. The majority of these costs were incurred in the clinical setting (87.7%). This is significantly higher than what was found in previous research [11], which showed an increase in costs of €1439. This difference might be explained by the more detailed registration and extensiveness of the databases included in the current study, which also provides more up-to-date information. Furthermore, it is notable that mental healthcare costs tend to increase during the 5 years leading up to the stroke event. Previous research [11] indicated that pre-stroke TIA might explain this trend, but our study could not confirm these findings. However, we did find that over 41% of the post-stroke mental healthcare costs were incurred by only 90 patients (25%). Additionally, increasing vascular complications leading up to the stroke event might be associated with depressive symptoms, resulting in higher mental healthcare consumption.

The results of the regression analyses show that age, number of hospitalization days and pre-stroke mental healthcare costs are significant predictors for post-stroke mental healthcare costs, and patients who lived with a partner incurred more mental healthcare costs both before and after they had a stroke. This is in line with previous research that showed that an age over 65 [13] and a long hospital stay [14, 15] were predictive factors for post-stroke costs. An explanation of why patients who live with a partner incur more costs might be because partners contribute to detecting symptoms of mental health problems in their significant others. In contrast with the current study, previous research found a variety of significant predictors of stroke-related costs [14–19]. This suggests careful consideration of underlying factors for predicting stroke-related mental healthcare costs. Furthermore, with regard to the upward trend of mental healthcare consumption preceding stroke in the Netherlands, the current study found similar results as those found in previous research [11]. However, despite the availability of more extensive information and variables from the data sources used, it remains unclear as to whether hypertension or diabetes, for example, are indicators for high mental healthcare costs pre- and post-stroke.

Post-stroke mental healthcare costs of €22,928 per patient for the 5 years following a stroke indicate significant costs when looking at all stroke patients on a national level. In the Netherlands, 83% of all clinical hospitalizations of stroke patients are due to first-ever strokes (corrected for mortality) [24]. Records show that 46,897 stroke patients were yearly discharged in 2010, resulting in 38,925 (83%) patients clinically hospitalized with a first-ever stroke [24]. According to our study results, 25,8% of this group used mental healthcare facilities accounting for approximately €6939 per stroke patient per year. This would result in €70 million stroke-related mental healthcare costs on a yearly basis. In 2010, the Dutch mental healthcare budget was €5.4 billion, which would amount to 1.3% of the annual mental healthcare budget in the Netherlands in 2010.

### Strengths and limitations

The linkage of three extensive, well-structured, prospective, databases providing information on stroke patients and mental healthcare consumption over a period of 10 years is considered to be a strength of this study. Another strength of this study is that the over fitting of the regression models was prevented by including variables based on the probability of the likelihood statistics [23]. Finally, the current study presents results of the most recent data available in combination with numerous possible predictors of stroke-related mental healthcare costs.

The current study was also subject to some limitations. First, this study is based on secondary data analysis using three data sources, which all contained patients from the same limited geographic area. Second, it did not include biological characteristics as possible predictors for stroke-related costs due to the unavailability of data. Previous research found several biological characteristics, such as heart failure, hyponatraemia, and atrial fibrillation to be significant predictors of stroke-related costs. Therefore, it would be interesting to include more possible predictors in future analyses.

## Conclusion

The main conclusion of the current study is that stroke patients have a significant mental healthcare comorbidity, leading to high post-stroke mental healthcare costs. Where previous research in 2001 found that strokes account for 1.3% of the total Dutch mental healthcare budget, our study results show equal findings in 2010. Although our study results and previous research show a significant relationship between stroke and mental healthcare consumption, information concerning the underlying factors responsible for these costs is inconclusive. Future research should focus on different predictors of stroke-related costs.

## Abbreviations

ICD: International Classification of Diseases
MA: Medical Administration
METC AZM/UM: Medical Ethics Committee Maastricht University Medical Center
MRS: Modified Ranking Scale
MSR: Maastricht Stroke Registry
MUMC: Maastricht University Medical Center
PCR: Psychiatric Case Registry
TIA: Transient Ischemic Attack
WHO: World Health Organization
